# Safety and Antitumor Activity of a Novel aCD25 Treg Depleter RG6292 as a Single Agent and in Combination with Atezolizumab in Patients with Solid Tumors

**DOI:** 10.1158/2767-9764.CRC-24-0638

**Published:** 2025-03-10

**Authors:** Valentina Gambardella, Michael Ong, Maria E. Rodriguez-Ruiz, Jean-Pascal Machiels, Miguel F. Sanmamed, Vladimir Galvao, Anna Spreafico, Daniel J. Renouf, Stephen J. Luen, Rachel Galot, Bernard Doger de Spéville, Emiliano Calvo, Aung Naing, Samira Curdt, Theresa Maria Kolben, Eva Rossmann, Tamara Tanos, Kevin Smart, Maria Amann, Yuying Xie, Linxinyu Xu, Enrique Gomez Alcaide, Nicolas Städler, Nicole Justies, Christophe Boetsch, Vaios Karanikas, Gabriel Schnetzler, Kristoffer S. Rohrberg

**Affiliations:** 1 Hospital Clinico Universitario de Valencia, INCLIVIA, Valencia, Spain.; 2 The Ottawa Hospital Cancer Centre, Ottawa, Canada.; 3 Department of Radiation Oncology, Clinica Universitaria de Navarra, Navarra, Spain.; 4 Department of Medical Oncology, Institut Roi Albert II, Cliniques universitaires Saint-Luc, and Institut de Recherche Clinique et Expérimentale, UCLouvain, Brussels, Belgium.; 5 Department of Medical Oncology, Clinica Universidad de Navarra, Pamplona, Spain.; 6 Vall d/Hebron Institute of Oncology (VHIO), Barcelona, Spain.; 7 Division of Medical Oncology and Hematology, Princess Margaret Cancer Centre, University Health Network, Toronto, Canada.; 8 British Columbia Cancer Agency - Vancouver, Vancouver, Canada.; 9 Division of Research, Peter MacCallum Cancer Centre, Melbourne, Australia.; 10 Sir Peter MacCallum Department of Oncology, The University of Melbourne, Melbourne, Australia.; 11 START Madrid Fundacion Jimenez Diaz, Early Fase Clinical Trial Unit, Madrid, Spain.; 12 START Madrid-CIOCC, Centro Integral Oncológico Clara Campal, Madrid, Spain.; 13 MD Anderson Cancer Center, Houston, Texas.; 14 Roche Innovation Center Munich, Roche Pharmaceutical Research and Development, Penzberg, Germany.; 15 Roche Innovation Center Basel, Roche Pharmaceutical Research and Early Development, Basel, Switzerland.; 16 Roche Innovation Centre Welwyn, Roche Pharmaceutical Research and Early Development, Welwyn, United Kingdom.; 17 Roche Innovation Center Zurich, Roche Pharmaceutical Research and Early Development, Schlieren, Switzerland.; 18 F. Hoffmann-La Roche Ltd., Mississauga, Canada.; 19 Department of Oncology, Copenhagen University Hospital, Copenhagen, Denmark.; 20 Department of Clinical Medicine, University of Copenhagen, Copenhagen, Denmark.

## Abstract

**Purpose:**

Therapeutic depletion of immunosuppressive regulatory T cells (Treg) may overcome resistance to cancer immunotherapies. RG6292 is an anti-CD25 antibody that preferentially depletes Tregs while preserving effector T-cell functions in preclinical models. The safety, pharmacokinetics, pharmacodynamics, and antitumor efficacy of selective Treg depletion by RG6292 administered as monotherapy or in combination with atezolizumab were evaluated in two phase I studies.

**Patients and Methods:**

Adult patients with advanced solid tumors were administered intravenous RG6292, given every 3 weeks alone (study 1: NCT04158583, *n* = 76) or with 1,200 mg atezolizumab every 3 weeks (study 2: NCT04642365, *n* = 49). Both studies included dose escalation and expansion parts to determine the maximum tolerated dose and recommended phase II dose.

**Results:**

RG6292 was well tolerated. Pruritus and rash were the most frequent adverse events and were manageable with supportive treatment. Serum RG6292 levels increased dose proportionally, independent of the atezolizumab combination. RG6292 induced a sustained dose-dependent depletion of peripheral Tregs with no apparent effect on other immune cells. Evidence of intratumoral Treg reduction (≥50% vs. baseline) was observed at RG6292 doses of 35 to 100 mg. The maximum tolerated dose was 165 mg every 3 weeks, and the recommended phase II dose was proposed as 70 mg every 3 weeks. Objective responses were limited to three partial responses in patients receiving RG6292 combined with atezolizumab.

**Conclusions:**

RG6292 induced a dose-dependent peripheral blood and measurable intratumoral Treg depletion in concordance with the proposed mode of action; however, clinical efficacy as a single agent or combined with atezolizumab was insufficient to warrant further exploration in this population.

**Significance:**

RG6292 (vopikitug) targets CD25 (IL-2Rα) and mediates regulatory T-cell depletion while not interfering with IL-2 signaling. Peripheral and intratumoral Treg depletion was shown in two phase I studies. However, RG6292 alone or in combination with atezolizumab was insufficient to reverse and rescue from established resistance mechanisms in solid tumors.

## Introduction

Despite recent advances in cancer immunotherapy (CIT)—particularly in the field of checkpoint inhibitors (CPI) that have led to improved survival rates—many patients fail to respond to treatment due to primary resistance, or they initially benefit but then progress due to secondary resistance (1). In many CD8-inflamed tumor types, elevated numbers of regulatory T cells (Treg) correlate with poor prognosis and reduced responsiveness to CPI therapy (2–4). Tregs can suppress antigen-specific antitumor immune responses and are hypothesized to be one of several mechanisms that can limit the effectiveness of CIT. Therefore, Tregs are considered an attractive clinical target aimed at improving the outcome of CIT (5–7).

CD25 (IL-2 receptor α-chain) is a cell surface marker expressed in several T-cell lineages, including high levels of expression on Tregs; it was proposed as a target for anticancer therapies more than 20 years ago (8, 9). However, prior explorations of the anti-human CD25 mAb daclizumab in combination with tumor-targeted vaccination strategies have failed to deliver clinical responses against solid tumors, likely due to bystander IL-2 receptor signaling blockade on effector T cells (Teff), which limits their antitumor activity (10, 11). Similarly, limited clinical activity was seen in an exploratory study with the chimeric anti-CD25 mAb basiliximab (12).

RG6292 (RO7296682, vopikitug) is the first anti-human CD25 antibody developed to specifically deplete human Tregs while preserving IL-2R STAT5 signaling and Teff activity. The fragment crystallizable (Fc) region of RG6292 is glycoengineered by afucosylation to mediate FcγR-dependent, CD25-targeted cell cytotoxicity (i.e., antibody-dependent cell cytotoxicity and antibody-dependent cellular phagocytosis). RG6292 binds with low monovalent affinity to the extracellular domain of the human CD25 antigen. However, a higher density of CD25 receptors favors bivalent binding and increases the apparent affinity of RG6292 to its target by more than 1,000-fold (avidity effect). Thus, RG6292 shows cell-type selectivity with preferential binding to T cells, which carry a high CD25 surface density, such as Tregs (13).

Two open-label, multicenter phase Ia/b studies were initiated to evaluate the safety, pharmacokinetic (PK) and pharmacodynamic (PD) properties, and antitumor activity of RG6292 when given alone (study 1, WP41188, NCT04158583) or in combination with the PD-L1 CPI atezolizumab (study 2, BP42595, NCT04642365) in patients with advanced solid tumors. As both studies had similar objectives, designs, and eligibility criteria and were conducted largely in parallel, they are jointly reported herein.

## Materials and Methods

### Patients

Adult patients (≥18 years) were included with histologically or cytologically confirmed RECIST version 1.1 measurable, locally advanced, or metastatic solid tumors [non–small cell lung cancer (NSCLC), head and neck squamous cell cancer (HNSCC), melanoma, ovarian cancer, triple-negative breast cancer (TNBC), or esophageal cancer] that had been previously treated and for which no standard or approved therapies were available. Patients had to have Eastern Cooperative Oncology Group performance status 0 or 1 and have adequate organ and bone marrow function. In study 2, the “Real wOrld PROgnostic (ROPRO) score” (14) was used to support the investigator with an objective assessment of the commonly used inclusion criteria “life expectancy, in the opinion of the investigator, of 12 weeks.” Patients with primary or uncontrolled metastases to the central nervous system were not eligible.

### Study design and objectives

Both studies were open-label, and RG6292 was given i.v. every 3 weeks in escalating doses either as monotherapy (study 1) or in combination with atezolizumab 1,200 mg every 3 weeks (study 2). Study 1 consisted of a dose-escalation stage (part A) and a dose-expansion stage (part B) to collect further biopsies and seek early signals of efficacy (Supplementary Fig. S1A). Study 2 (Supplementary Fig. S1B) was planned to include three parts: part I as dose escalation of RG6292 in combination with atezolizumab, part II as dose expansion in patients with acquired CPI resistance (i.e., documented tumor response or at least stable disease for at least 4 months prior to relapse) and a histologically confirmed inflamed tumor immune phenotype, and part III as tumor-specific expansion cohorts in patients who failed on prior CPI therapy. Opening of part III was dependent on achieving predefined efficacy gates in either study 1 or parts I and II of study 2 [objective response rate (ORR) target ≥15–20%].

All patients were treated until disease progression, unacceptable toxicities, withdrawal of consent, or withdrawal of the participant by the investigator. Patients were allowed to continue treatment with the investigational medicine(s) for a maximum of 24 months.

The primary objective in both studies was to characterize the safety, tolerability, maximum tolerated dose (MTD), and/or recommended phase II dose (RP2D) of RG6292 alone or in combination with atezolizumab. Secondary objectives were to investigate the PK profile of RG6292, evaluate the antidrug immune response after treatment, and assess the preliminary antitumor activity of RG6292. Additional exploratory objectives involved the evaluation of the relationship between RG6292 exposure and PD biomarkers including peripheral and tissue (i.e., tumor) T-cell phenotype and numbers (absolute and ratios).

### Rationale for dose selection

A starting dose of 0.3 mg i.v. was selected for study 1 (15). This dose was expected to be (i) subpharmacological (<10% CD25 receptor occupancy at Cmax and <5% average Treg depletion over 3 weeks), (ii) safe (below the threshold to trigger strong IL-6 release in whole-blood assay and >100-fold exposure margin over No-Observed-Adverse-Effect-Level (NOAEL) from a Good-Laboratory-Practice (GLP) toxicity study), and (iii) close to the minimal pharmacologically active dose (mPAD), expected to be reached within three cohorts. In study 2, a backbone therapy with the approved dosing schedule for atezolizumab 1,200 mg i.v. every 3 weeks was used (16), whereas a starting dose of 0.3 mg for RG6292 was considered appropriate and safe.

### Study drug administration

Patients were given RG6292 every 3 weeks by i.v. infusion as ascending flat doses with a starting dose of 0.3 mg. RG6292 was administered over 4 hours at the first infusion and thereafter in shorter applications if tolerated. In study 2, atezolizumab 1,200 mg was administered every 3 weeks additionally prior to RG6292 for all infusions. Premedication was allowed at the investigator’s discretion, and in case of infusion-related reactions, antihistamines and/or antipyretics and/or antiemetics were recommended.

### Assessments

Demographics and medical history were recorded at screening (up to 4 weeks prior to the first dose). Physical assessments including vital signs, Eastern Cooperative Oncology Group (ECOG) performance status, and assessment of organ function (including hematologic, cardiac, and kidney) were performed at screening and on treatment until the last safety follow-up visit. Screening for autoantibodies was performed at baseline, at cycles 2 and 3, and every 6 cycles thereafter. Pregnancy testing (urine or serum) was mandated in women of childbearing potential prior to each treatment, with results available before dosing. Optional nutritional assessment and stool sampling at screening and every 12 weeks or in case of colitis were conducted for further microbiome analysis.

### Dose limiting toxicity and safety

Safety assessment was performed by investigators according to the NCI Common Terminology Criteria for Adverse Events version 5.0 and classified as related or unrelated to study drug administration. A dose-limiting toxicity (DLT) was defined as a clinically significant adverse event (AE; typically ≥grade 3) or significant laboratory abnormality related to RG6292 occurring during the DLT assessment period between the first administration and until 7 days after the second administration of RG6292. In addition, any other RG6292-related toxicity considered significant enough to be qualified as a DLT in the opinion of the investigator after discussion with the sponsor was deemed a DLT. During the dose escalation phase, participants who withdrew before the end of the DLT period, for reasons other than DLTs, and participants who did not receive two doses of RG6292 were to be replaced to ensure that at least three DLT-evaluable participants were assessed prior to moving to the next dose level.

For classification purposes, lower-level terms were assigned by the sponsor to the original terms using the most up-to-date version of the Medical Dictionary for Regulatory Activities (MedDRA) 25.1 terminology for AEs. In this study, disease progression was not to be reported as an AE.

### PK and biomarker assessments

Blood samples were collected to quantify RG6292 serum concentrations before and after infusion, as well as at several time points after the first infusion and during cycle 4. In all other cycles, samples were taken before and 3 hours after infusion for RG6292 serum concentration measurement. In study 2, additional blood samples for serum concentration measurement of atezolizumab were taken 30 minutes after atezolizumab infusion during cycles 1 and 4. Serum concentrations of RG6292 and atezolizumab were measured using validated ELISA methods. The validated lower limit of quantification for the RG6292 assay was 19.1 ng/mL in human serum.

Individual and mean serum RG6292 and atezolizumab concentrations were determined, and nonlinear mixed-effect modeling was used to analyze the concentration–time data (Supplementary Fig. S2). Population and individual PK parameters were estimated, and the influence of various covariates (such as age, gender, and body weight) on these parameters was investigated in an exploratory way. Secondary PK parameters (such as Cmax and AUC) were derived from the model for each participant included in the PK analysis.

Testing for antidrug antibody (ADA) against RG6292 and atezolizumab was conducted using predose blood samples at each cycle, at the end of the treatment, and at the safety follow-up. The RG6292 ADA sample analysis was performed with a validated bridging ELISA. The ELISA was conducted at a constant serum concentration of 1.0% accuracy.

Blood samples were collected for PD assessment at every cycle. Whole-blood samples were used for assessment of changes in the characteristics of lineage (T-cells, NK cells, monocytes, Tregs, B cells, etc.), activation (such as HLA-DR), differentiation (such as CD152 and PD-1), and proliferation (Ki67) of peripheral blood immune cells by flow cytometry. Serum and/or plasma samples were used to analyze cytokines and inflammation markers (such as TNFɑ, IFN-ɣ, IL-8, CXCL10, IL-2, IL-6, IL-10, and soluble CD25).

Archival tumor tissue or, when not available, fresh tumor tissue samples were taken for baseline assessment. In study 1, on-treatment biopsies were taken once a dose was reached in which a reduction to 25% of baseline peripheral Treg level and/or a fourfold increase of peripheral Teff/Treg ratio was observed in at least 50% of patients. Such biopsies were collected, provided they were clinically feasible, lesions were accessible, and the participant consented. In study 2, on-treatment biopsies were planned only for parts II and III.

Intratumoral content of CD8^+^ T cells and FOXP3+ cells was assessed by IHC. Immunophenotype was characterized by the number and location of CD8^+^ tumor-infiltrating lymphocytes and defined as inflamed, excluded, and desert (17).

### Tumor response

Tumor response was evaluated by investigators according to RECIST version 1.1 (18). Tumor lesions were assessed at screening every 8 weeks after the first dose (i.e., C1D1) for the first year and then every 12 weeks thereafter until disease progression. Additional MRI or CT scans were performed at the end of the treatment (28 days after the last dose) and at the safety follow-up (135 days after the last dose). Survival follow-up was performed every 3 months thereafter.

### Statistical analysis and sample size justification

No formal statistical model and no formal hypothesis testing were planned in these studies. Dose escalation in both studies applied a modified continual reassessment method with an escalation with overdose control design, and dose levels selected were based on the occurrence of DLTs (19, 20). The MTD was defined as the dose with the highest probability that the DLT rate was within the target of 20% to 33% and a relatively low probability (<25%) that the DLT rate was above 33%. At least three DLT-evaluable participants were enrolled at each dose level.

In study 1, the maximum total number of participants in the dose escalation portions of part A was estimated to be approximately 90 DLT-evaluable participants on an every 3-week schedule. For study 1 part B, up to a maximum of 50 patients with melanoma, NSCLC, or HNSCC were planned to be enrolled to obtain approximately 20 evaluable paired tumor biopsy samples. This would provide 80% confidence that 50% of participants will achieve a reduction to 25% of baseline Treg levels in the tumor.

In study 2, the maximum sample size for part I (dose escalation) was 60 patients, and approximately 40 patients with melanoma, NSCLC, or HNSCC and secondary CPI resistance were planned to be enrolled in part II, aiming to have at least 30 response-evaluable participants with an inflamed phenotype confirmed in the fresh baseline biopsy.

For part III of study 2, a sample size of 20 response-evaluable patients was planned per cohort. However, part III was not opened.

### Ethics

The protocols and their subsequent amendments were approved by the local Institutional Review Board at the participating centers. All patients provided written informed consent prior to any study-related procedures, and the study was performed in accordance with the Declaration of Helsinki and International Conference for Harmonization of Good Clinical Practice Guidelines. Study 1 was registered on ClinicalTrials.gov as NCT04158583, and study 2 was registered as NCT04642365. Both studies were sponsored and funded by F. Hoffmann-La Roche.

### Data availability

Researchers may request access to individual patient-level clinical data through Vivli (https://vivli.org/ourmember/roche/). For up-to-date details on Roche’s Global Policy on the Sharing of Clinical Study Information and how to request access to related clinical study documents, please refer to https://go.roche.com/data_sharing. Anonymized records for individual patients across more than one data source external to Roche cannot, and should not, be linked due to a potential increase in the risk of patient reidentification. All other data are available from the corresponding author upon request.

## Results

### Patient characteristics

Study 1 (WP41188) enrolled 76 patients between December 9, 2019, and October 27, 2021, at 11 study centers across five countries. The last patient, last visit corresponding to the clinical cutoff date used for the analyses presented herein was July 21, 2022. The sponsor decided to halt recruitment following part A, as a higher likelihood for efficacy was expected if RG6292 was combined with atezolizumab, which was evaluated in study 2.

Study 2 (BP42595) enrolled 49 patients (*n* = 46 in part I and *n* = 3 in part II) between January 4, 2021, and October 3, 2022, at 10 study sites across six countries. On October 3, 2022, the sponsor decided to terminate the study early due to recruitment challenges for part II and as the totality of data generated indicated a low likelihood of achieving the targeted efficacy required to open part III. Consequently, part III was not opened, and the last patient, last visit corresponding to the clinical cutoff date for study 2 was January 4, 2024.

All enrolled patients of both studies were considered in the safety, DLT, PK, and PD analyses. One patient from study 2, part I was excluded from the efficacy analysis, as inclusion criteria were not met.

Patient disposition of the two studies is summarized in Table 1.

**Table 1:** Patient disposition

	Study 1 (RG6292 monotherapy)	Study 2 (RG6292 plus atezolizumab)
Screened	95	61
Enrolled	76	49
Age (years)		
Median (min–max)	58.5 (34–80)	60.0 (31–80)
Gender		
Male	33 (43.4%)	26 (53.1%)
Female	43 (56.6%)	23 (46.9%)
Race		
Asian	6 (7.9%)	2 (4.1%)
White	70 (92.1%)	46 (93.9%)
Black	0 (0%)	1 (2%)
ECOG		
0	32 (42.1%)	20 (40.8%)
1	44 (57.9%)	29 (59.2%)
Prior lines of therapy		
Median (min–max)	3 (1–8)	3 (1–6)
Prior radiotherapy	47 (61.8%)	26 (53.1%)
Prior CPI	45 (59.2%)	33 (67.3%)
Tumor		
TNBC	8 (10.5%)	2 (4.1%)
Esophageal cancer	7 (9.2%)	3 (6.1%)
HNSCC	14 (18.4%)	7 (14.3%)
Melanoma	19 (25%)	15 (30.6%)
NSCLC	7 (9.2%)	10 (20.4%)
Ovarian cancer	21 (27.6%)	12 (24.5%)

Abbreviations: ECOG, Eastern Cooperative Oncology Group; TNBC, triple-negative breast cancer.

### Dose escalation and DLTs

In study 1, RG6292 doses were escalated in nine cohorts (0.3, 1, 2, 6, 18, 35, 70, 100, and 165 mg). Six patients experienced seven DLTs, including rash, papular rash, and maculopapular rash, whereas one patient had two DLTs (aspartate transaminase elevation and alanine aminotransferase elevation; see Table 2). The MTD was defined at 165 mg. An additional backfill cohort at 20 mg was opened after reaching the MTD to obtain additional tumor biopsies.

**Table 2:** Summary of key outcome parameters of study 1 (WP41188; RG6292 every 3 weeks)

Cohort	1	2	3	4	5	6	7	8	9	10^a^
RG6292 dose	0.3 mg	1 mg	2 mg	6 mg	18 mg	35 mg	70 mg	100 mg	165 mg	20 mg
*n*	5	5	6	14	8	5	15	6	6	6
DLTs, *n*					1 (rash)		2 (Rash) 1 (ALT↑ + AST↑)		1 (Rash)	1 (Rash)
Cmax (μg/mL)	0.0718 (26.9)	0.256 (16.1)	0.505 (34.3)	1.79 (57.5)	5.38 (31.3)	9.80 (29.1)	22.4 (25.9)	38.2 (26.7)	75.9 (46.2)	7.46 (30.6)
AUC_tau_ (h × μg/mL)	12.6 (46.0)	46.9 (8.7)	104 (33.4)	280 (29.5)	980 (41.1)	1,750 (23.2)	3,940 (29.6)	5,920 (38.8)	10,600 (34.4)	1,040 (22.7)
% with blood Treg depletion to ≤25% of baseline	0% (0/4)	25% (1/4)	20% (2/5)	82% (9/11)	100% (7/7)	100% (4/4)	100% (13/13)	100% (5/5)	100% (6/6)	100% (5/5)
% with intratumoral Treg depletion ≤50% of baseline	na	na	na	1/7	0/1	2/2	4/10^b^	1/3	0/3	1/3

Abbreviations: ALT, alanine aminotransferase; AST, aspartate transaminase; na, not applicable.

a Backfill cohort for additional on-treatment biopsy collection after MTD has been reached.

b One sample had <1 Treg/mm^2^ at baseline.

In study 2, RG6292 doses were escalated across seven dose cohorts (0.3, 1.5, 9, 20, 40, 80, and 160 mg). Three patients reported one DLT each: immune system disorder (*n* = 1) and maculopapular rash (*n* = 2; Table 3). The MTD was not reached up to dosing of 160 mg of RG6292 in combination with the standard dose of atezolizumab.

**Table 3:** Summary of key outcome parameters of study 2 (BP42595; RG6292 plus atezolizumab 1,200 mg every 3 weeks)

Cohort	1	2	3	4	5	6	7	Part II
RG6292 dose	0.3 mg	1.5 mg	9 mg	20 mg	40 mg	80 mg	160 mg	70 mg
*n*	6	6	7	7	7	6	7	3
DLTs, *n*				1 (Immune disorder)		2 (Rash)		
Cmax (μg/mL)	0.0921 (54.1)	0.555 (22.2)	3.18 (19.3)	8.23 (32.8)	12.7 (40.9)	27.3 (34.5)	61.7 (17.5)	na
AUC_tau_ (h×μg/mL)	14.3 (89.3)	83.7 (50.2)	507 (43.9)	1,050 (26.9)	2,100 (50.8)	3,860 (22.3)	9,890 (23.8)	na
% with blood Treg depletion to ≤25% of baseline	17% (1/6)	20% (1/5)	100% (5/5)	83% (5/6)	100% (7/7)	83% (5/6)	100% (3/3)	na

Abbreviation: na, not applicable.

A summary of DLTs, as well as key PK and PD measures by dose cohort, is provided in Table 2 (study 1) and Table 3 (study 2).

### Safety

An overview of key safety results in both studies is provided in Table 4.

**Table 4:** Safety summary

*n* (%)	Study 1 (RG6292 monotherapy; *n* = 76)		Study 2 (RG6292 plus atezolizumab; *n* = 49)	
	All	Related	All	Related
AE (any grade)	75 (98.7)	60 (78.9)	48 (98.0)	42 (85.7)
AE grade 3–4	31 (40.8)	14 (18.4)	25 (49.0)	10 (20.4)
Serious AE	20 (26.3)	4 (5.3)	21 (42.9)	8 (16.3)
AE leading to dose modification/interruption	19 (25.0)	11 (14.5)	16 (32.7)	12 (24.5)
AE leading to treatment discontinuation	2 (2.6)	2 (2.6)	5 (10.2)	3 (6.1)

In study 1, 75 (98.7%) patients reported a total of 561 AEs, of which AEs in 60 (78.9%) patients were considered related to the study drug by the investigator. AEs related to study treatment reported at a frequency of >10% by preferred term were pruritus (31.6%), rash (27.6%), and maculopapular rash and asthenia (10.5% each). No grade 5 AE was reported in this study. All skin events ≥grade 3 were seen in patients receiving 18 mg dosage and above. Most skin toxicity events were grade 1 or 2 at the highest severity. Grade 3 events were reported in nine patients who all received the study drug at 18 mg and above. The majority of patients with skin toxicities were treated with topical or low-dose systemic corticosteroids, and the events had recovered or were recovering at the time of analysis. However, skin events led to dose interruption in ten patients, dose reduction in two patients, and study drug withdrawal in one patient. Overall, 45 (59.2%) patients died mainly due to disease progression or disease relapse.

In study 2, 48 (98.0%) patients reported a total of 515 AEs, of which AEs in 42 (85.7%) patients were considered related to the study drug by the investigator. The most frequently reported drug-related AEs by preferred term occurring in at least 10% of patients were pruritus (40.8%), rash (34.7%), maculopapular rash (22.4%), asthenia (20.4%), and fatigue (12.2%). These AEs were judged to be related to both study drugs, with the exception of one event each of maculopapular rash and pruritus, and two events of rash, which were reported to be related to RG6292 only. Again, most skin toxicity events were grade 1 or 2 in severity. Grade 3 events were reported in four participants who received ≥9.0 mg RG6292. The majority of participants with skin toxicities were treated with corticosteroids, while skin toxicities led to drug interruption in eight patients and study drug withdrawal in one patient. Most skin events had recovered or were recovering at the time of analysis. One grade 5 serious AE (infection) was reported with an initial severity of grade 3, which was considered not related to study treatment. Overall, 31 (63.3%) patients died in the study, and 30 of these died due to progressive disease or disease relapse.

The RG6292 dose was modified in 25% and 32.7% of patients in study 1 and study 2, respectively, suggesting no or limited synergistic toxicity when combining RG6292 with atezolizumab.

### Antitumor activity

In study 1, a total of 22 patients had stable disease as the best overall response (BOR), whereas none of the 76 patients achieved an objective response (complete or partial response), resulting in an ORR of 0% [95% confidence interval (CI): 0.0, 4.7] and a disease control rate (DCR) of 28.9% (95% CI: 19.1, 40.5). Seventy-three (96.1%) patients had a progression event, and the estimated median progression-free survival (PFS) was 1.9 months (95% CI: 1.8, 1.9). Forty-five (59.2%) patients died, and the estimated median overall survival was 8.6 months (95% CI: 5.1, 11.0).

Among the 48 efficacy-evaluable patients in study 2, three achieved a BOR of partial response, resulting in an ORR of 6.4% (95% CI: 0.0, 14.1). The three patients with tumor responses were enrolled in part I: one patient with ovarian cancer (40 mg cohort), one patient with NSCLC (40 mg cohort), and one patient with esophageal cancer (80 mg cohort). Nineteen patients had stable disease as BOR, resulting in a DCR of 45.8% (95% CI: 31.4, 60.8). The patients with ovarian and esophageal cancer whose tumors responded have not been treated with anti–PD-1/anti–PD-L1 treatment prior to enrolling in this study. The patient with NSCLC was treated with approved and investigational anti-PD-1 treatment 9 and 11 months prior to enrolling in this study, respectively.

Forty-four (91.7%) patients had a progression event, and the estimated median PFS was 2.3 months (95% CI: 1.9, 3.5), whereas 31 (64.6%) patients died, and the estimated median overall survival was 8.6 months (95% CI: 7.0, 11.6). Spider plots showing change from baseline in the sum of diameters in target lesions over time are provided in Supplementary Fig. S3A and S3B.

### PK and PD

Serum concentrations of RG6292 increased rapidly and reached peak levels (Cmax) around 4 to 6 hours after starting the infusion. The serum exposures of RG6292 increased with dose, independent of atezolizumab combination (Tables 2 and 3). No apparent signs of target-mediated drug disposition could be observed. The mean elimination half-life ranged from 8 to 13 days. The combination of atezolizumab did not significantly change these parameters, as observed in study 2.

None of the patients in study 1 and only 2 out of 49 (4.6%) patients in study 2 had positive postbaseline ADA titer values for RG6292.

A receptor occupancy assay was developed to measure target engagement because receptor occupancy and CD4^+^ Treg killing correlated strongly with preclinical *in vivo* and *ex vivo* experiments (15). However, the assay applied to the clinical samples did not perform as expected and could not be used for data analysis and interpretation.

**Figure 1:**
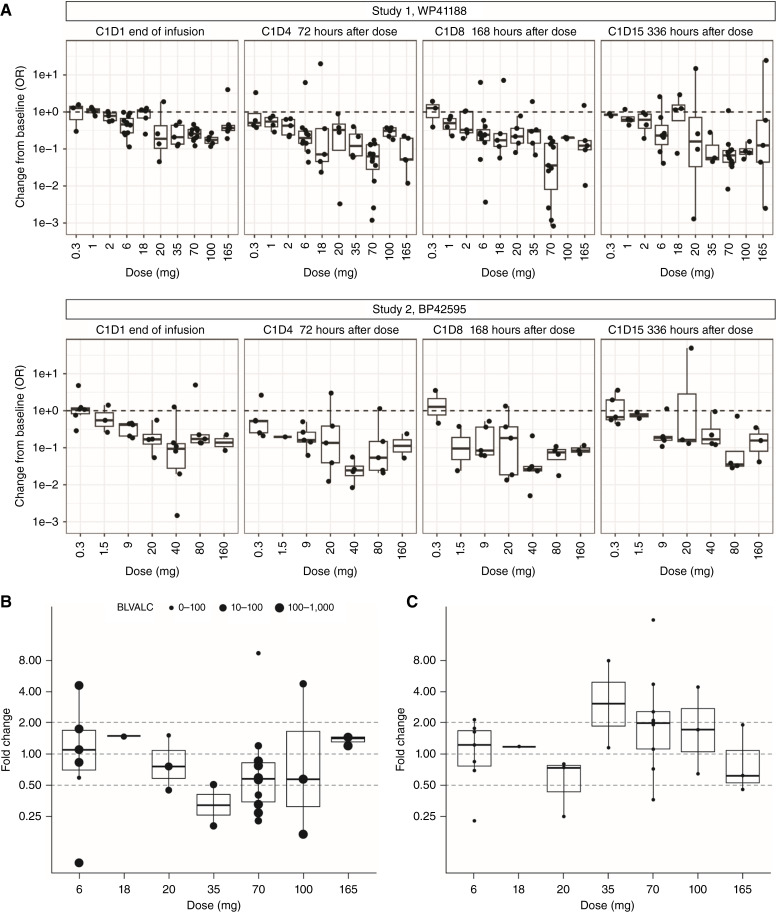
**A,** A single infusion of RG6292 results in sustained peripheral Treg depletion in a dose-dependent manner and independent of atezolizumab administration. **B,** RG6292 leads to intratumoral Treg reduction. The size of the dots reflects the number of Tregs/mm^2^ at baseline. Note that one sample of cohort 7 (70 mg) had <1 Treg/mm^2^ at baseline. **C,** Change in Teff/Treg ratio. Graphs show fold change from baseline in the ratio of CD8^+^ cells to FOXP3^+^ cells for each individual paired biopsy analyzed based on IHC of intratumoral CD8^+^ and FOXP3^+^-stained cells, respectively.

Independent of combination with atezolizumab, treatment with RG6292 induced a sustained dose-dependent peripheral blood Treg depletion at doses >0.3 mg, and the majority of patients showed peripheral Treg depletion to <25% of baseline level at doses ≥6 mg (Tables 2 and 3). A single infusion of RG6292 results in sustained peripheral Treg depletion in a dose-dependent manner and independent of atezolizumab administration (Fig. 1A), whereas less than twofold mean change of absolute Teff numbers was seen (Supplementary Fig. S4A–S4C). No effect was observed on other peripheral immune cell populations such as NK cells or B cells. Levels of soluble CD25 increased marginally in participants treated at doses >18 mg of RG6292, whereas dose-independent, low-level increases of TNFɑ, CXCL10, and transient changes in IL-6, IL-8, and IFN-ɣ were observed. The majority (>95% of samples) of IL-2 measurements remained below the lower limit of quantification of the assay (1.08 pg/mL).

A total of 29 paired fresh tumor biopsies collected at baseline and on treatment at cycle 2, day 8 were available, with doses ranging from 6 to 165 mg in study 1. The quantitative assessment of FOXP3 and CD8 IHC staining suggests that RG6292 induces measurable changes in FOXP3^+^ cell numbers within tumors when comparing on-treatment biopsies to matched baseline biopsies. This effect was observed predominantly in patients receiving doses of 35 to 100 mg although no formal statistical analyses were performed (Table 2; Fig. 1B and C). No major changes in immunophenotype were seen in paired biopsies from 22 participants analyzed: Upon treatment, seven paired biopsies showed a conversion into a more inflamed phenotype, twelve did not change, and three moved toward a less inflamed phenotype. No consistent gene expression alterations or immune signatures could be observed when comparing baseline and on-treatment biopsies in RNA sequencing analysis. However, a trending increase in immunosuppressive macrophages was noted. No tumor biopsies were available from study 2.

Exploratory analyses of paired biopsies of unaffected healthy skin did not reveal any clear pattern of Treg depletion. Microbiota analyses (Shannon alpha diversity and Bray–Curtis beta diversity) of stool samples did not show any relevant changes between baseline and C4D15. Both the skin and microbiota analyses were confounded by the limited sample number available.

**Figure 2:**
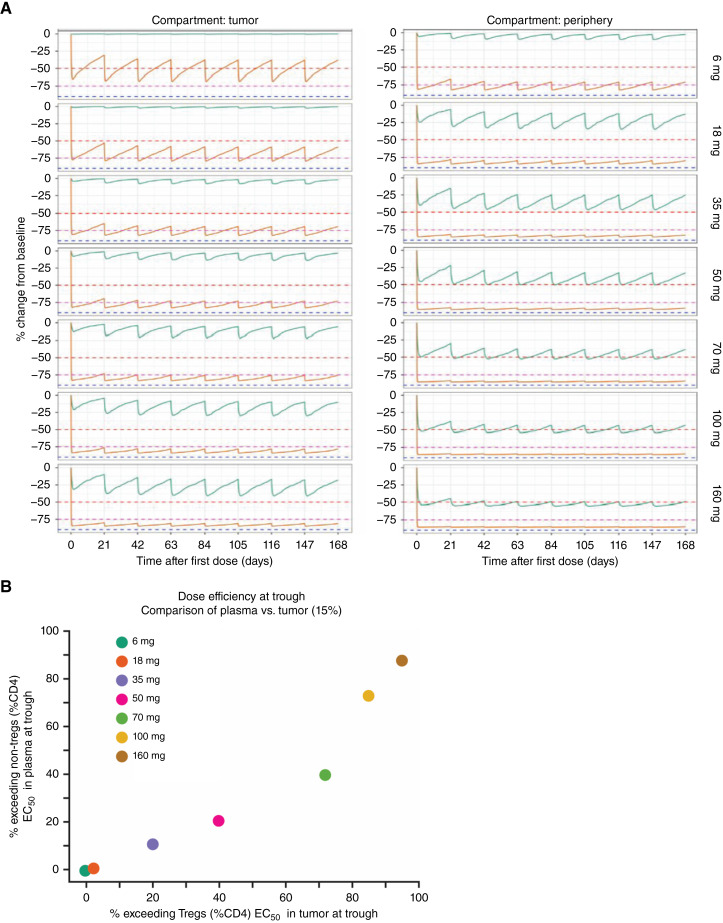
**A,** Simulation plots: The PK/PD model was used to simulate both RG6292 and FOXP3^+^ Treg levels following a range of doses. The simulations were based on the population parameters from the PK/PD model and were summarized (median) by analyte (green, non-Tregs; orange, Tregs) and dose. The solid lines represent the median, the shaded areas span the 5th and 95th percentiles, and the horizontal dashed lines represent 50% (red), 75% (magenta), and 90% (blue) change from baseline. **B,** Therapeutic window plot: fraction of simulated patients with RG6292 trough concentration in plasma above the EC_50_ for non-Tregs vs. the fraction of simulated patients with RG6292 trough concentration in tumor above the EC_50_ for Tregs, colored by dose.

A population PK/PD modeling approach was applied to Tregs and Teffs in the periphery to predict the RG6292 effects in the tumor microenvironment (Supplementary Fig. S2). The PK/PD relationships observed and characterized in the periphery for all cell subpopulations were adjusted, assuming a tumor uptake factor of 15%. The recommended phase II dose was proposed as 70 mg every 3 weeks to favor intratumoral Treg depletion while minimizing the potential effect on non-Tregs in the periphery (Fig. 2A). It was predicted that at steady-state trough, this would lead to 72% of patients with an RG6292 concentration above the Treg (%CD4) EC_50_ in the tumor (positive effect) and 40% of patients with an RG6292 concentration above the non-Treg (%CD4) EC_50_ in plasma (potentially negative effect; Fig. 2B).

## Discussion

In this study, we present the results from two multicenter, dose-finding, and dose-expansion studies of RG6292 (vopikitug), the first anti-human CD25 antibody developed to preferentially deplete human Tregs without affecting essential IL-2 signaling (13). RG6292 demonstrated linear PK behavior in doses up to the MTD of 165 mg every 3 weeks. Pruritus and rash were the most frequent AEs and could be managed with topical steroids or a short course of low-dose systemic steroids (≤10 mg/day prednisone). Combination with atezolizumab 1,200 mg every 3 weeks was consistent with the safety profile of the individual components, without evidence of synergistic toxicity.

Although RG6292 induced a dose-dependent peripheral blood and measurable intratumoral Treg depletion in concordance with the proposed mode of action (i.e., antibody-dependent cell cytotoxicity and antibody-dependent cellular phagocytosis; ref. 13), the clinical efficacy as a single agent or in combination with atezolizumab was unfortunately insufficient to further explore this antibody in tumor-specific patient cohorts (Supplementary Table S1). However, some patients experienced long-term disease stabilization lasting >6 months.

The lack of clinical activity of the anti-CD25 mAb daclizumab given in combination with cancer vaccines to patients with melanoma or ovarian cancer was hypothesized to be linked to the detrimental effects of the IL-2 signaling blockade on cytotoxic Teffs (10, 11). Clinical investigation of the CD25-targeted antibody-drug conjugate camidanlumab tesirine in solid tumors has recently been terminated as efficacy signals in combination with pembrolizumab were insufficient despite signals of immunomodulatory activity (NCT03621982; ref. 21). Similarly, another Treg-depleting approach using a CCR4-targeted mAb mogamulizumab demonstrated strong peripheral and intratumoral depletion of the CCR4^+^ subset of Tregs yet with limited clinical activity (22, 23). However, concomitant depletion of CCR4-expressing NK cells and central memory CD8^+^ T cells might impede the antitumor therapeutic effect of mogamulizumab in solid tumors (24).

In contrast, RG6292 was specifically designed to bind with a different epitope of CD25 and, therefore, preserve the essential IL-2–mediated signaling and Teff activity (13). In our study, RG6292 treatment did not seem to affect the number or functionality of intratumoral CD8 T cells, nor was any effect on PD-L1 expression evident. This is also supported by an experiment conducted as part of the preclinical safety evaluation: An *in vitro* Epstein–Barr virus (EBV) reactivation assay using whole blood from healthy donors was performed, in which RG6292 was tested against daclizumab at clinically relevant exposures. As a result, daclizumab triggered a dose-dependent reactivation of EBV, which was comparable with the positive control cyclosporine. In contrast, RG6292 did not reactivate EBV, suggesting maintained functionality of Teff memory cells.

Due to the relatively low ORR of 6% in study 2, we compared the clinical efficacy of atezolizumab in combination with RG6292 with recent phase I/II studies in which atezolizumab was combined with other investigational immunomodulators (25–27). However, there was no relevant difference with respect to our observed DCR and median PFS of 49% and 2.3 months, respectively. This suggests that RG6292 did not negatively affect the established clinical activity of atezolizumab.

Extensive biomarker assessments of blood and tumor biopsies were performed and did not reveal a substantial impact on non-Tregs after treatment. However, a trending increase in immunosuppressive macrophages could point toward homeostatic substitution by alternative resistance mechanisms (28).

Despite the strong correlation of Tregs with poor prognosis and reduced responsiveness to CPI therapy in solid tumors (2–4), it remains elusive whether intratumoral Treg depletion can activate an immune response and translate into clinical efficacy. To the best of our knowledge, there is currently no target with prognostic or predictive cutoff values defined in solid tumors. Initially, we aimed for a Treg reduction to 25% of baseline value or a fourfold increase of the Teff/Treg ratio. Following treatment with RG6292, we saw approximately a twofold reduction in about half of the patients with available paired biopsies in the target dose range. This is attributable to a treatment effect, as a change of at least twofold is required to be considered biologically meaningful to eliminate any interpretation confounded by assay variability (29). Nevertheless, the RG6292-mediated intratumoral Treg depletion was less pronounced than what has been observed in the blood, and contributing factors remain unknown.

As high intratumoral Tregs remain a key prognostic factor for clinical outcomes in many tumors, earlier intervention to prevent Treg-mediated resistance or alternative combinations might be considered. Additionally, various other Treg-depleting strategies, such as anti-CCR8 mAbs designed to selectively eliminate intratumoral Tregs in solid tumors, are currently under clinical evaluation (30–32). These novel approaches might provide further insights into the clinical relevance of Treg-depleting strategies.

## Supplementary Material

Table S1. Representativeness of Study Participants.

Supplementary Figure 1A and 1B Figure S1A. Study design - Study 1 (WP41188; NCT04158583). Figure S1B. Study design - Study 2 (BP42595; NCT04642365).

Supplementary Figure 2 Figure S2. Population PK/PD modeling approach: The PK/PD model was used to simulate both RG6292 and FOXP3+ Treg levels, following a range of doses. The model which best describes the data is a model where previously defined drug levels (from the PK model) stimulate the loss of CD25+ cells from the plasma, in a Michaelis-Menten manner. The concentrations of RG6282 and the cell count of the respective T cells were modeled in a simultaneous manner. Proportional error was used for both PK and PD. PK/PD modeling utilized the measured plasma concentrations of different CD25+ cell types, like Treg cells or CD4+CD25+ following IV infusion administration of RG6292. 

Supplementary Figure 3 Figure S3. Spider plots showing percentage change from baseline in sum of diameters in target lesions for efficacy-evaluable patients. (A) Study 1 (doses 18 mg and above) and (B) Study 2 (doses 20 mg and above).

Supplementary Figure 4 Figure S4. (A) The levels of peripheral CD8 T cells (CD45+CD3+CD8+) after treatment with RG6292 or (B) RG62962 in combination with atezolizumab show less than 2-fold mean change of absolute T-cell numbers with intra patient variations (odds ratio of CD8+ cells are calculated with regards to CD3+ cells). (C) Quantitative analysis of on-treatments biopsies to matched baseline biopsies shows no systematic change of CD8+ T cells.
