# Supplementary material for: Safety and Antitumor Activity of a Novel aCD25 Treg Depleter RG6292 as a Single Agent and in Combination with Atezolizumab in Patients with Solid Tumors
Source: Cancer Res Commun. 2025 Mar 10;5(3):422–32. doi: 10.1158/2767-9764.CRC-24-0638 (PMC11891644; doi:10.1158/2767-9764.CRC-24-0638)
Supplement: Table S1. — Representativeness of Study Participants. [file crc-24-0638_table_s1.suppst1.docx]

**Table S1.** Representativeness of Study Participants

| Representativeness of Study Participants | |
| --- | --- |
| Cancer type(s)/subtype(s)/stage(s)/condition | This study enrolled patients with locally advanced or metastatic solid tumors (non-small cell lung cancer [NSCLC], head and neck squamous cell cancer [HNSCC], melanoma, ovarian cancer, triple-negative breast cancer [TNBC], or esophageal cancer). The patients had previously been treated prior to the study, and no approved therapies were available. These tumors were considered “inflamed,” and provided the scientific rationale for this cancer immunotherapy approach. Therefore, patients with less immunogenic tumors (e.g. CRC) were not included. |
| Considerations related to: | |
| Sex | Overall, the two studies enrolled patients with an equal sex distribution across the different tumors, except for the biologically driven difference for TNBC and ovarian cancer. |
| Age | The median age of the enrolled population was approximately 60 years old. This is in line with the general epidemiology of the studied cancer types. |
| Race/ethnicity | The population enrolled in the two studies were mostly of Caucasian ethnicity (>90%). This population is not representative of the ethnicity distribution of the relevant cancer types. |
| Geography | Study 1 enrolled participants at a total of 11 sites in five countries in Europe, Canada and Australia. Study 2 enrolled participants at a total of 10 sites in Europe, Canada, US and Australia. The data is therefore only relevant to the populations from which patients were enrolled. |
| Other considerations | The selection of participants and outcome measures of these small phase 1 studies were driven by tumor biology and general clinical conditions. |
| Overall representativeness of this study | The selected patient population are relevant for the overall study aim. The sex of the enrolled patients was equally distributed across different tumor types, except for TNBC and ovarian cancer. The median age of the enrolled patients was representative of the general age relevant to the studied cancer types. |
