# Supplementary material for: Safety and Antitumor Activity of a Novel aCD25 Treg Depleter RG6292 as a Single Agent and in Combination with Atezolizumab in Patients with Solid Tumors
Source: Cancer Res Commun. 2025 Mar 10;5(3):422–32. doi: 10.1158/2767-9764.CRC-24-0638 (PMC11891644; doi:10.1158/2767-9764.CRC-24-0638)
Supplement: Supplementary Figure 1A and 1B — Figure S1A. Study design - Study 1 (WP41188; NCT04158583). Figure S1B. Study design - Study 2 (BP42595; NCT04642365). [file crc-24-0638_supplementary_figure_1a_and_1b_suppsf1a-1b.pdf]

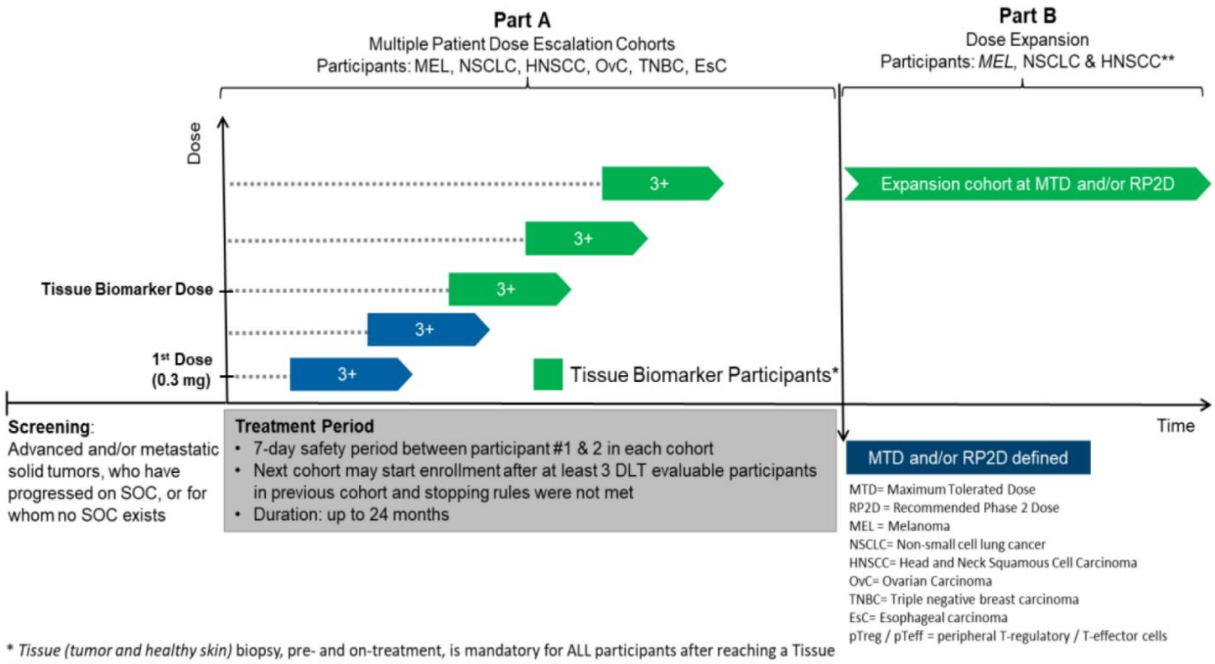

* *Tissue (tumor and healthy skin)* biopsy, pre- and on-treatment, is mandatory for ALL participants after reaching a Tissue Biomarker Dose provided tumor lesion is accessible and biopsy feasible. Tissue Biomarker Dose is defined as the effective dose that induces either a reduction to 25% of baseline pTreg level and/or a ≥4 fold increase of pTeff/pTreg ratio as compared to baseline and/or a dose where clinical response (CR or PR) is observed.

**In the expansion cohort a maximum of 50 participants across the three indications will be enrolled, with approximately 20 participants having matched paired biopsies (i.e. biopsies from same patient at both time points).

SOC=Standard of Care; DLT=Dose Limiting Toxicities.

Figure S1A. *Study design - Study 1 (WP41188; NCT04158583).*

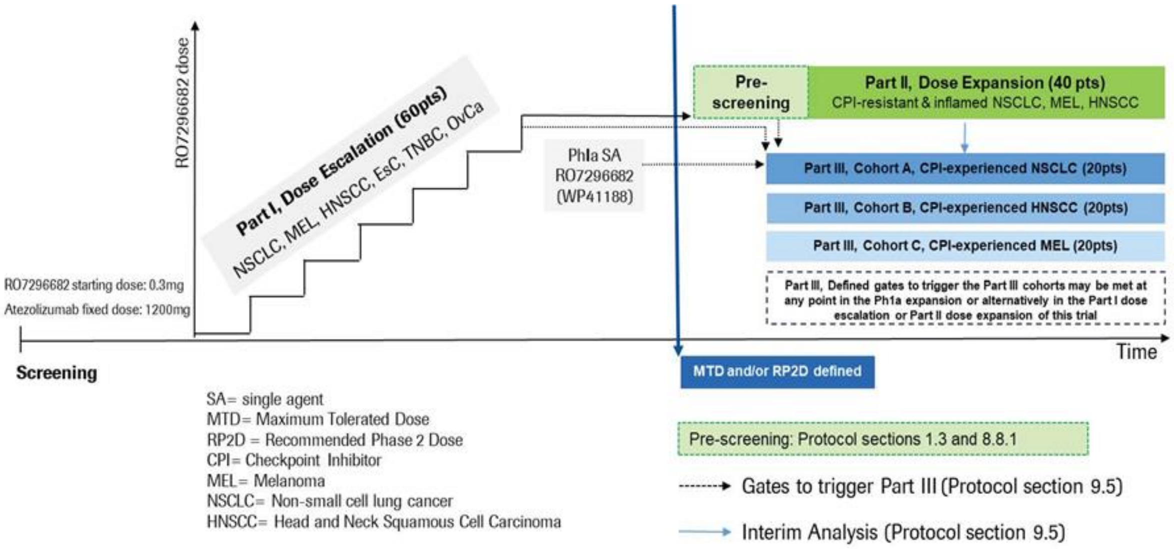

Figure S1B. *Study design - Study 2 (BP42595; NCT04642365).*
