# Supplementary material for: Safety and Antitumor Activity of a Novel aCD25 Treg Depleter RG6292 as a Single Agent and in Combination with Atezolizumab in Patients with Solid Tumors
Source: Cancer Res Commun. 2025 Mar 10;5(3):422–32. doi: 10.1158/2767-9764.CRC-24-0638 (PMC11891644; doi:10.1158/2767-9764.CRC-24-0638)
Supplement: Supplementary Figure 2 — Figure S2. Population PK/PD modeling approach: The PK/PD model was used to simulate both RG6292 and FOXP3+ Treg levels, following a range of doses. The model which best describes the data is a model where previously defined drug levels (from the PK model) stimulate the loss of CD25+ cells from the plasma, in a Michaelis-Menten manner. The concentrations of RG6282 and the cell count of the respective T cells were modeled in a simultaneous manner. Proportional error was used for both PK and PD. PK/PD modeling utilized the measured plasma concentrations of different CD25+ cell types, like Treg cells or CD4+CD25+ following IV infusion administration of RG6292. [file crc-24-0638_supplementary_figure_2_suppsf2.pdf]

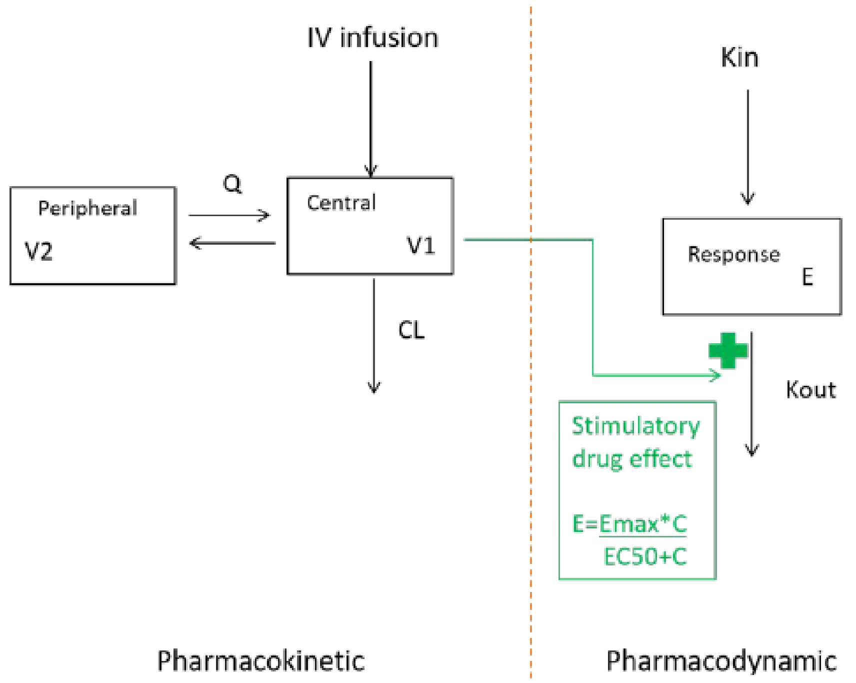


E = Treg cells
Kin = Production rate
Emax = Maximum effect
EC50 = Concentration for 50% of the Treg response
C = RG6292 concentration
Kout = Rate of loss,
defined as Kin/E0 where E0 = Baseline

Non-Tregs (%CD4)
Tregs (%CD4)

***Supplementary Figure 2****. Population PK/PD modeling approach: The PK/PD model was used to simulate both RG6292 and FOXP3+ Treg levels, following a range of doses.*

The model which best describes the data is a model where previously defined drug levels (from the PK model) stimulate the loss of CD25+ cells from the plasma, in a Michaelis-Menten manner. The concentrations of RG6282 and the cell count of the respective T cells were modeled in a simultaneous manner. Proportional error was used for both PK and PD. PK/PD modeling utilized the measured plasma concentrations of different CD25+ cell types, like Treg cells or CD4+CD25+ following IV infusion administration of RG6292.
