# Supplementary material for: Safety and Antitumor Activity of a Novel aCD25 Treg Depleter RG6292 as a Single Agent and in Combination with Atezolizumab in Patients with Solid Tumors
Source: Cancer Res Commun. 2025 Mar 10;5(3):422–32. doi: 10.1158/2767-9764.CRC-24-0638 (PMC11891644; doi:10.1158/2767-9764.CRC-24-0638)
Supplement: Supplementary Figure 3 — Figure S3. Spider plots showing percentage change from baseline in sum of diameters in target lesions for efficacy-evaluable patients. (A) Study 1 (doses 18 mg and above) and (B) Study 2 (doses 20 mg and above). [file crc-24-0638_supplementary_figure_3_suppsf3.pdf]

A. Study 1, WP41188

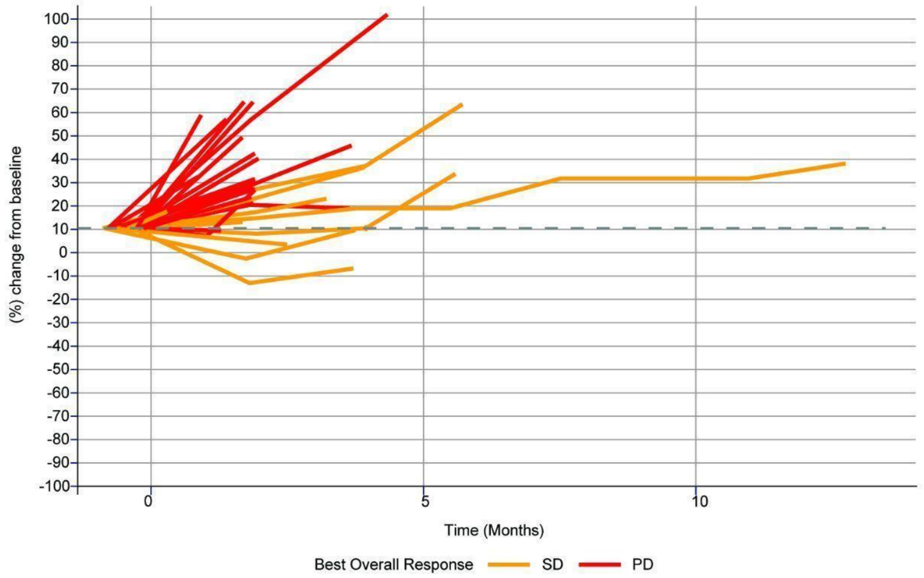

B. Study 2, BP42595

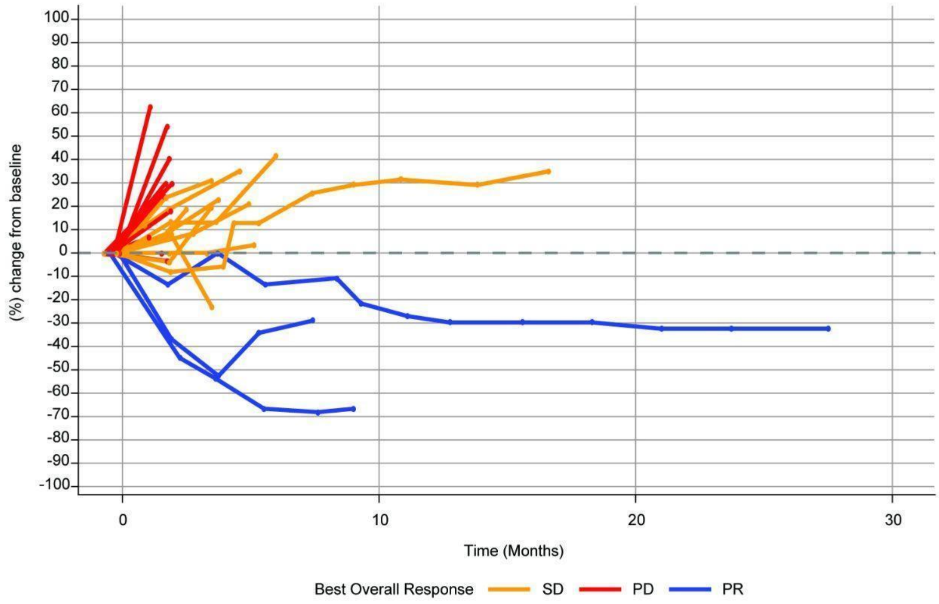

Figure S3. *Spider plots showing percentage change from baseline in sum of diameters in target lesions for efficacy-evaluable patients.* ***(A)*** *Study 1 (doses 18 mg and above) and* ***(B)*** *Study 2 (doses 20 mg and above).*
