# Supplementary material for: Safety and Antitumor Activity of a Novel aCD25 Treg Depleter RG6292 as a Single Agent and in Combination with Atezolizumab in Patients with Solid Tumors
Source: Cancer Res Commun. 2025 Mar 10;5(3):422–32. doi: 10.1158/2767-9764.CRC-24-0638 (PMC11891644; doi:10.1158/2767-9764.CRC-24-0638)
Supplement: Supplementary Figure 4 — Figure S4. (A) The levels of peripheral CD8 T cells (CD45+CD3+CD8+) after treatment with RG6292 or (B) RG62962 in combination with atezolizumab show less than 2-fold mean change of absolute T-cell numbers with intra patient variations (odds ratio of CD8+ cells are calculated with regards to CD3+ cells). (C) Quantitative analysis of on-treatments biopsies to matched baseline biopsies shows no systematic change of CD8+ T cells. [file crc-24-0638_supplementary_figure_4_suppsf4.pdf]

A)

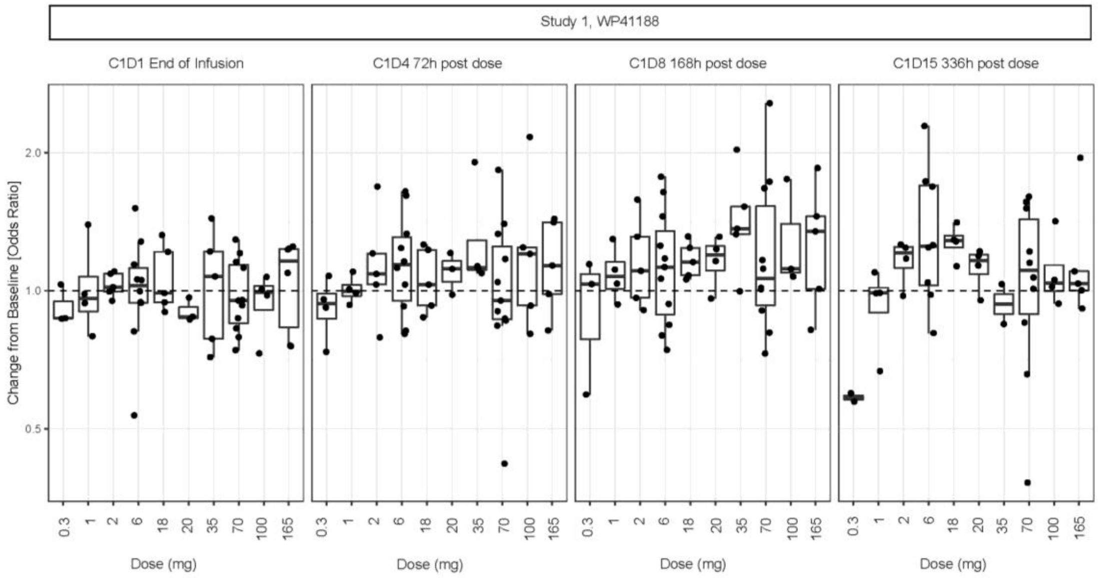

B)

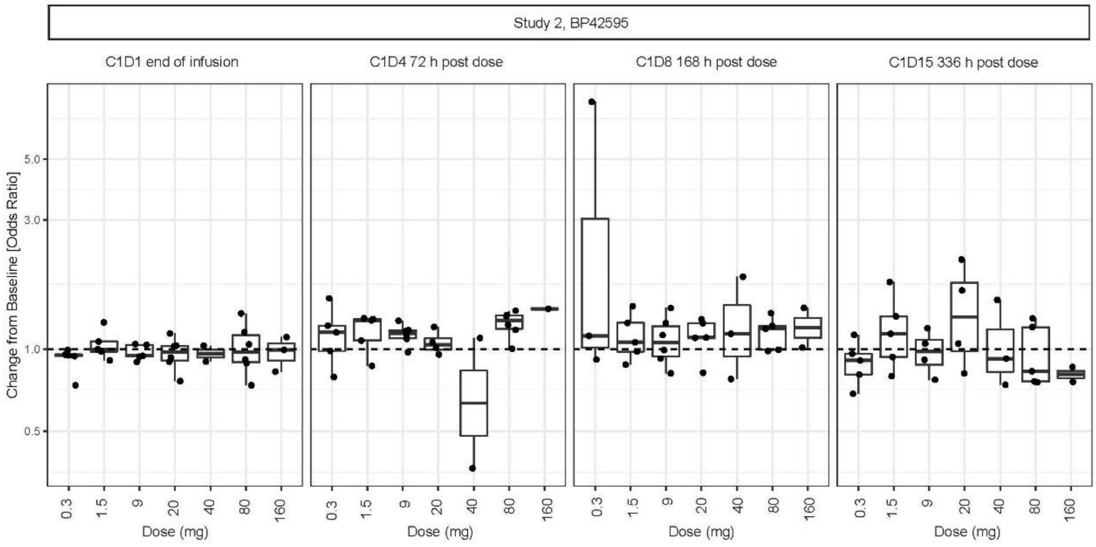

C)

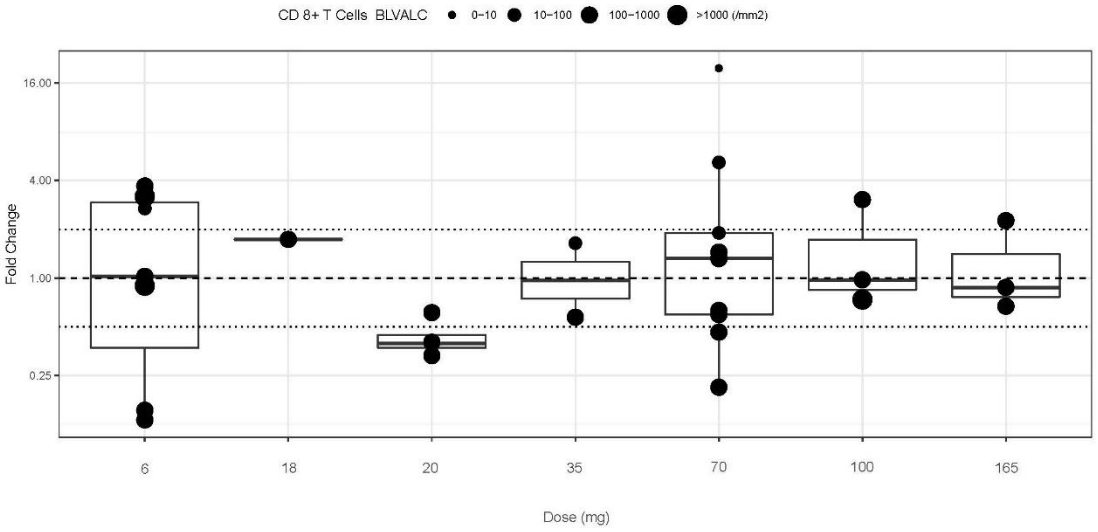

Figure S4. (A) *The levels of peripheral CD8 T cells (CD45+CD3+CD8+) after treatment with RG6292 or* ***(B)*** *RG62962 in combination with atezolizumab show less than 2-fold mean change of absolute T-cell numbers with intra patient variations (odds ratio of CD8+ cells are calculated with regards to CD3+ cells).* ***(C)*** *Quantitative analysis of on-treatments biopsies to matched baseline biopsies shows no systematic change of CD8+ T cells.*
